# Supplementary material for: Adaptive Pseudo Text Augmentation for Noise-Robust Text-to-Image Person Re-Identification
Source: Sensors (Basel). 2025 Nov 24;25(23):7157. doi: 10.3390/s25237157 (PMC12694070; doi:10.3390/s25237157)
Supplement: Supplementary file 1 [file sensors-25-07157-s001.zip › sensors-3956759-supplementary.pdf]

Supplementary Material

1. Multi-seed statistical results

Table S1. Retrieval performance under three random seeds (mean ± std). The bolds are the best performance values.

Dataset	Method	R-1	R-5	R-10	mAP	mINP
ICFG-PEDES	RDE	67.72 ± 0.53	82.38 ± 0.45	87.09 ± 0.36	41.96 ± 0.51	7.86 ± 0.13
	Ours	68.20 ± 0.34	83.19 ± 0.29	88.23 ± 0.42	41.36 ± 0.45	7.89 ± 0.18
RSTPReid	RDE	65.34 ± 0.49	84.99 ± 0.43	90.12 ± 0.26	51.04 ± 0.18	29.03± 0.21
	Ours	66.24 ± 0.26	85.89 ± 0.38	91.37 ± 0.29	52.35 ± 0.26	29.09 ± 0.24

2. TAL hyperparameter sensitivity

Figure S1. Variation in performance with different m and τ in RSTPReid

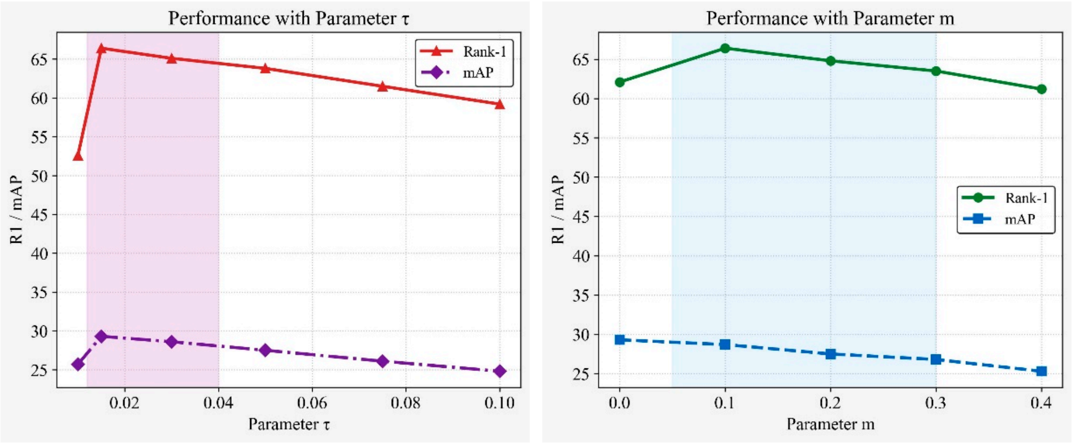

3. Sensitivity to batch size

Table S2. Effect of batch size in ICFG-PEDES dataset. The bolds are the best performance values.

Batch size	R-1	R-5	R-10	mAP	mINP
32	66.42	81.23	87.19	39.86	7.53
64	68.20	83.19	88.23	41.36	7.89
128	67.69	81.84	88.52	39.94	7.03
